# Association Between Low Ankle-Brachial Index and High Common Carotid Artery Intima-Media Thickness in Ischemic Stroke

**DOI:** 10.7759/cureus.83246

**Published:** 2025-04-30

**Authors:** Rohan Kelkar, Shivashankar K N, Prakashini K, Raveena Kelkar, Nishad A Barve

**Affiliations:** 1 Internal Medicine, Kasturba Medical College, Manipal, Udupi, IND; 2 Radiology, Kasturba Medical College, Manipal, Udupi, IND; 3 Internal Medicine, Wentworth-Douglass Hospital, Dover, USA; 4 Internal Medicine, Lowell General Hospital, Lowell, USA

**Keywords:** ankle brachial index, carotid intima-media thickness, cerebrovascular accident (stroke), cross-sectional studies, peripheral arterial diseases

## Abstract

Introduction

Stroke is a significant contributor to death and illness globally. Primary prevention of stroke involves aggressive control of risk factors like hypertension, diabetes, dyslipidemia, and smoking. Studies have been conducted to search for surrogate markers of increased ischemic stroke risk. Increased carotid artery intima-media thickness (CIMT) is a recognized indicator of increased stroke risk. Recently, ankle brachial index (ABI) has been increasingly studied for its predictive value in ischemic stroke. ABI is easier to calculate bedside using a simple hand-held Doppler, while CIMT involves the use of expensive equipment and the services of an expert radiologist. The goal of the present study was to investigate the relation between ABI and CIMT in patients with ischemic stroke.

Methods

The current cross-sectional study was conducted at a tertiary care hospital in South India. After obtaining informed consent, 50 patients with an ischemic stroke who met the inclusion and exclusion criteria were recruited in our study. ABI and CIMT were measured. Patients were subdivided into groups of low (≤0.9) and high ABI (>0.9) and low (<0.9 mm) and high CIMT (≥0.9 mm).

Results

The mean CIMT in the present study was 1.025 ± 0.63 mm. The mean ABI of the study population was 0.989 ± 0.22. There was a negative correlation between CIMT and ABI (r = -0.367), with a p value < 0.01. Further, there was an association between higher CIMT values and lower ABI values in patients with ischemic stroke.

Conclusion

There is an inverse relationship between ABI and CIMT in ischemic stroke. A low ABI was associated with a high CIMT value in ischemic stroke.

## Introduction

Stroke is a significant contributor to mortality and morbidity in India and globally [1,2]. Stroke can be either hemorrhagic or ischemic, of which the latter is predominant [3]. Atherosclerosis of intracranial and extracranial vessels is a significant cause of ischemic stroke [4].

Atherosclerosis is a chronic and systemic process that affects the vascular system of our body and is a result of inflammatory and immune mechanisms [5]. Pathophysiologically, atherosclerosis is a very important cause of stroke, myocardial infarction (MI), and peripheral arterial disease (PAD). The factors associated with atherosclerosis as well as stroke are diastolic or systolic hypertension, dyslipidemia, diabetes mellitus, cigarette smoking, and consumption of alcohol [6,7].

The ankle-brachial index (ABI) is a non-intrusive technique to screen for early atherosclerosis [8]. It is calculated by dividing the systolic blood pressure at the ankle (dorsalis pedis or posterior tibial artery) by the systolic blood pressure at the brachial artery. PAD is diagnosed when the ABI is less than 0.9. A low ABI has been linked in studies to an increased chance of developing cardiovascular diseases like coronary artery disease and stroke [9].

Additionally, carotid artery intima-media thickness (CIMT) is an important indicator of atherosclerosis and predicts cerebrovascular disease due to ischemia [10]. A high CIMT has been linked to a higher risk of stroke and coronary artery disease [11,12]. Carotid ultrasonography is an efficient, highly reproducible, and comparatively inexpensive method for the evaluation of atherosclerotic changes by assessing the intima-media thickness (IMT) or plaques in the carotids. Carotid plaques and increased carotid IMT are very important predictors of cardiovascular disease or ischemic stroke [13-15].

Research has proposed a predictive role for ABI in predicting future ischemic stroke [16-18]. Identifying subclinical PAD can be a valuable prognostic indicator to determine those who are at higher risk of experiencing an ischemic stroke. Previous studies have shown a significant relation between CIMT and ABI in diabetic subjects [5]. If any numerical relation is found between ABI and CIMT, ABI can be used to predict ischemic stroke. ABI is simple, reliable, inexpensive, and quick to perform as compared to Doppler-CIMT.

Numerous studies have reported an association between a low ABI and high CIMT and increased risk of cardiovascular disorders, including stroke [10,19,20]. But the inter-relationship between ABI and CIMT in ischemic stroke has been underexplored, particularly in the Indian population.

We conducted a hospital-based, cross-sectional observational study in patients presenting with ischemic stroke at a tertiary care hospital in South India to investigate the relationship between ABI and CIMT. Specifically, we aimed to determine whether ABI, a simple and cost-effective bedside tool, is inversely associated with CIMT, a well-established marker of subclinical atherosclerosis. By evaluating this relationship, the study also explores the potential of ABI as a predictive marker for stroke risk, especially in settings with limited access to vascular imaging.

## Materials and methods

The current cross-sectional study was conducted at a tertiary care hospital in South India from November 2016 to July 2018 and adhered to the Helsinki Declaration of 1975. Prior to the study's commencement, institutional ethics committee approval was obtained (Approval Number: IEC 778/2016).

Calculation of sample size

The sample size was estimated based on detecting a moderate correlation (r = -0.4) between ABI and CIMT, as reported in earlier literature, such as the study by Yoon et al., where a similar inverse relationship was found (r = -0.378) [21]. The sample size was determined using the following formula for correlation studies:

$N = \left(\frac{Z\alpha + Z\beta}{C}\right)^2 + 3$

Where *Z_α_* is the standard normal deviate for a two-tailed significance level of 0.05 (1.96), and *Z*_*β*_is the standard normal deviate for a power of 80% (0.84) [22].

The constant *C* is derived from the Fisher Z transformation of the correlation coefficient *r*:

$C = 0.5 \times \ln(1 + r / 1 - r)$

Substituting the expected correlation of -0.4 into the equation yields *C* = 0.4236. Plugging all values into the formula:

$N = \left(\frac{1.96 + 0.840}{0.4236}\right)^2 + 3 \approx 47$.

Accordingly, the minimum required sample size was calculated to be approximately 47 participants. To account for potential dropouts and ensure sufficient power, a total of 50 ischemic stroke patients were included in the study.

A total of 50 patients who presented to the hospital with an ischemic stroke (cardioembolic and hemorrhagic strokes were excluded) and who met the requirements for inclusion and exclusion were enrolled in our study after acquisition of written informed consent. All patients had their medical history taken and underwent clinical examination. Magnetic resonance imaging (MRI), including diffusion-weighted imaging, was used to confirm the presence of an ischemic stroke. Routine biochemical investigations were done as per standard of care.

Measurement of ankle brachial index (ABI)

Ankle brachial index was calculated using a manual sphygmomanometer with a 12 cm wide cuff and a Vascular Doppler 320 (Vcomin Technology Limited, Guangdong, China). Patients were instructed to rest in a supine position for at least 10 minutes before measurements were taken. Each foot’s ABI was determined independently by dividing the higher ankle systolic pressure (posterior tibial or dorsalis pedis artery) by the greater systolic pressure of the two brachial arteries, as shown in Table 1. ABI ≤ 0.9 was taken as a low ABI. ABI > 1.3 was considered abnormal, as it is suggestive of non-compressible vessels. The lower value of the left and right ABI was taken as the ABI for that patient [23].

**Table 1:** Calculation of left and right ankle brachial index. ABI: ankle brachial index.

Calculation of ABI	
Right ABI	Higher right ankle pressure/higher arm pressure
Left ABI	Higher left ankle pressure/higher arm pressure

Measurement of common carotid artery intima-media thickness (CIMT)

**Figure 1:**
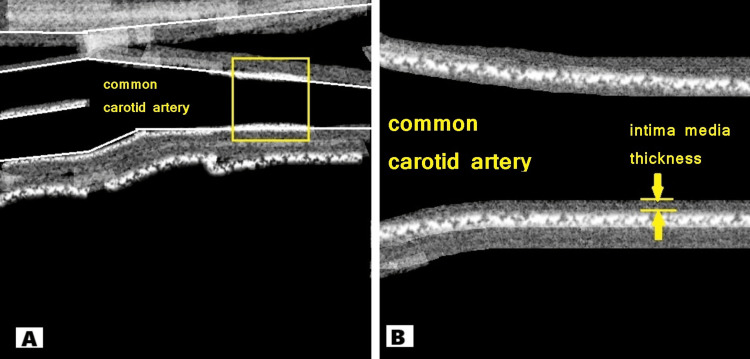
Illustration showing the measurement of common carotid artery intima-media thickness using ultrasonography. (A) Selection of the common carotid artery area for measurement. (B) Magnified image showing intima-media thickness.

CIMT was estimated by a specialist radiologist using a Philips HD 11XE ultrasound machine (Philips Healthcare, Bothell, Washington). The patient was placed in the supine position, and the head was turned opposite to the side being examined. B-mode (bright-mode) ultrasonography was performed using a linear phased-array transducer with a fundamental frequency of ~7 MHz to obtain images showing both the anterior and posterior walls of the common carotid artery, as illustrated in Figure 1. CIMT was measured as the interval between the lumen-intima and media-adventitia junctions of the carotid artery. CIMT measurements were obtained on the posterior wall proximal to the carotid bifurcation. To get the CIMT, six values, three on each side, were acquired and averaged. While measuring the CIMT, areas of the common carotid artery that had a plaque or calcified lesion protruding into the lumen were avoided [24].

Previous studies have demonstrated that the risk for stroke and myocardial infarction increases when the CIMT is ≥ 0.9 mm, which was also used as the cut-off value for abnormal CIMT in our study [10,25].

Inclusion criteria

Patients > 18 years of age with ischemic stroke, primary or recurrent, diagnosed using magnetic resonance imaging (MRI), were included in the study.

Exclusion criteria

Patients with hemorrhagic stroke, peripheral vascular disease, upper/lower limb deformity, limb amputation, revascularization of the carotid or lower limb arteries using percutaneous or surgical means, patients on therapy with statins for > one year, and patients with ABI > 1.4 were excluded from the study.

Statistical analysis

Statistical analysis was performed using IBM SPSS Statistics for Windows, version 20 (IBM Corp., Armonk, New York). Descriptive statistics were reported as mean ± standard deviation (SD) for continuous variables and as frequencies and percentages for categorical variables. Normality of data distribution was assessed using the Shapiro-Wilk test. The Pearson correlation coefficient (r) was used to analyze the relationship between ABI, CIMT, and other continuous variables in the study population after adjusting for potential confounders. An independent t-test was used to compare continuous variables between the two groups having low and normal ABI. Results with a p-value less than 0.05 were considered statistically significant. The chi-square test was utilized to test for the association between categorical data.

## Results

A total of 50 ischemic stroke patients were analyzed. The basic features of the study population are enumerated in Table 2.

**Table 2:** Basic features of the study population. ABI: ankle-brachial index.

Variable	Sub-variable	ABI ≤ 0.9	ABI > 0.9	Total	Percentage
Sex	Males	13	17	30	60
	Females	8	12	20	40
Age (mean)	-	62.14 ± 22.08	60.13 ± 22.5	-	-
Diabetes	No	10	20	30	60
	Yes	11	9	20	40
Hypertension	No	8	13	21	42
	Yes	13	16	29	58
Smoking	No	13	25	38	76
	Yes	8	4	12	24
Alcohol	No	15	25	40	80
	Yes	6	4	10	20

In our study, there were 30 males (60%) and 20 females (40%). There were 21 patients (42%) who had low ABI (≤0.9), and 29 (58%) had normal ABI. The mean ABI was 0.98948.

The mean CIMT for the study population was 1.025 ± 0.63 mm. There were 32 patients (64%) who had CIMT ≥0.9 mm, and 18 (36%) had CIMT <0.9 mm. The mean CIMT for the low ABI group was 1.13 ± 0.68 mm while that for the normal ABI group was 0.94 ± 0.55 mm. Using an independent t-test, a significant difference was found between the mean CIMT values of the two groups (p = 0.039).

**Figure 2:**
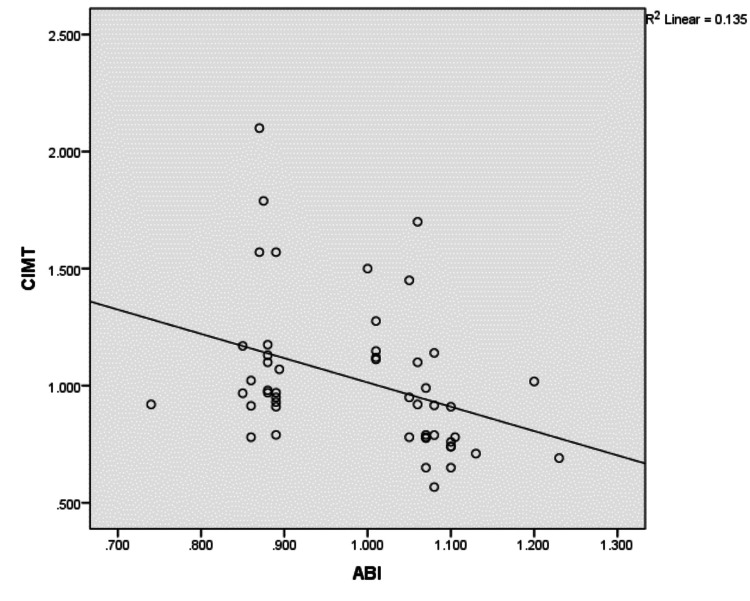
Inverse correlation between ABI and CIMT. r = -0.367; p-value < 0.01. ABI: ankle brachial index; CIMT: common carotid artery intima-media thickness; R2: coefficient of determination.

After adjusting for potential confounders, there was an inverse correlation present between ABI and CIMT values with a correlation coefficient of -0.367 (p < 0.01), as shown in Figure 2.

Using the chi-square test, a significant association (p-value = 0.034) was found between low ABI (ABI ≤ 0.9) and high CIMT (CIMT ≥ 0.9 mm) in our study population, as shown in Table 3.

**Table 3:** Chi-square test for the association between ABI and CIMT. ABI: ankle brachial index; CIMT: common carotid artery intima-media thickness.

Mean CIMT groups	ABI > 0.9	ABI ≤ 0.9	p-value
Mean CIMT < 0.9 mm	14	4	0.034
Mean CIMT ≥ 0.9 mm	15	17	

There was no significant difference in the mean CIMT and mean ABI values among males and females. Similarly, there was no significant difference in the mean CIMT and mean ABI values among smokers, hypertensives, diabetics, alcohol users, and those not having these risk factors.

## Discussion

Previous research has shown that ABI and CIMT are indicators of atherosclerosis and have been linked to an increased risk of cardiovascular disease [10]. Also, there are reports that the ABI value in patients with ischemic stroke was lower than that of a normal population, and the CIMT value was significantly higher than normal subjects [9].

In this study, analyzing the risk factors, most patients were males, and a majority were >45 years of age. This is expected as age and male sex are established risk factors for stroke [26]. Both the groups, i.e., ABI ≤ 0.9 and ABI > 0.9, were comparable with respect to the risk factors of age, sex distribution, hypertension, diabetes, and risk factors like smoking and alcohol.

The prevalence of CIMT ≥ 0.9 mm was 64% in ischemic stroke. The mean CIMT in our study was 1.025 ± 0.63 mm. The mean CIMT values in studies by Sahoo et al. and Das et al. were 0.798 mm and 0.849 ± 0.196 mm, respectively [27,28]. We got higher CIMT values, which can be due to the fact that we had a higher prevalence of diabetics and hypertensives in the study population.

The prevalence of abnormal ABI in our study was 42%. The present study found an inverse linear correlation between ABI and CIMT values. CIMT and ABI showed an inverse correlation (r = -0.367), which was statistically significant (p < 0.01). This is similar to a study by Yoon et al., which found a weak inverse correlation (r = -0.378) between ABI and CIMT [13]. Brasileiro et al. found a similar negative correlation (r = -0.235) in patients with high cardiovascular risk [29].

CIMT among those with low ABI was significantly higher than those with normal ABI. A statistically significant difference (p = 0.039) was observed in the mean CIMT between the two groups (1.13 ± 0.68 mm vs. 0.94 ± 0.55 mm). 

The chi-square test was employed to test for association between CIMT ≥ 0.9 mm and ABI ≤ 0.9, which was significant (p = 0.034). This means that a low ABI was associated with a high CIMT among patients with ischemic stroke.

Our study did have some limitations. The small sample size limits the generalizability of the findings to a larger population. The exclusion of patients with peripheral vascular disease, limb deformities, and prolonged statin therapy further limits the applicability to broader stroke populations. Also, while ABI and CIMT are useful in screening for atherosclerosis-related strokes, they are ineffective for strokes caused due to cardiac embolism, arterial dissection, hemorrhage, and venous thrombosis. ABI and CIMT were not serially assessed, which can help to assess the efficacy of treatment.

There is no consensus in the literature regarding the normal value of carotid intima-media thickness, and different studies use different thresholds, leading to variability in interpretation. The absence of controls from the general population in our study hindered the evaluation of various stages of atherosclerosis. The cross-sectional study design limits the ability to identify a causal relationship between ABI, CIMT, and ischemic stroke. Therefore, ABI cannot be considered a replacement for CIMT in clinical practice at this stage. Instead, ABI may serve as a complementary tool, especially in resource-limited settings where CIMT measurement is not readily available.

## Conclusions

This study highlights the significant prevalence of peripheral artery disease (ABI ≤ 0.9) and increased carotid artery intima-media thickness (CIMT ≥ 0.9 mm) among patients with ischemic stroke. Our findings indicate that a low ABI is associated with higher CIMT values, suggesting that both are useful indicators of atherosclerosis and increased ischemic stroke risk. The inverse relationship between ABI and CIMT suggests that these non-invasive measures can serve as complementary tools for identifying individuals at higher stroke risk.

The take-home message is that early screening using ABI and CIMT can identify individuals having subclinical atherosclerosis and increased ischemic stroke risk. These cost-effective, simple techniques can be valuable tools in stroke prevention and risk stratification in clinical practice.
